# Supplementary material for: Mitochondrial electron transport chain, ceramide and Coenzyme Q are linked in a pathway that drives insulin resistance in skeletal muscle
Source: bioRxiv. 2023 Sep 19:2023.03.10.532020. Originally published 2023 Mar 11. Preprint. [Version 2] doi: 10.1101/2023.03.10.532020 (PMC10028964; doi:10.1101/2023.03.10.532020)
Supplement: 1 — Figure S1 A) Mitochondrial enriched fraction from L6-myotubes. Calnexin was used as a marker of endoplasmic reticulum. OXPHOS: oxidative phosphorylation system. Cholesterol (B), Diacylglycerol (DAGs) (C), Sphingosine-1 phosphate (S1P) (D), and Sphingosine (SPH) (E) abundance in L6 myotubes exposed to different compounds (as indicated). Lipid abundance was determined by lipidomics and normalised against protein concentration. N = 4, Mean ± S.E.M. ***p< 0.001, ****p< 0.0001. F) Concentration of endogenous ceramide in L6-HA-GLUT4 myotubes treated for 24 h with EtOH (control) or different concentrations of Saclac. N = 3, mean ± S.E.M. *p<0.05. **p< 0.01, ****p< 0.0001. G-H) L6-HA-GLUT4 myotubes were serum-starved after EtOH (Control) or Saclac (10 μM) treatment (for 24 h) and acute insulin (Ins) was added where indicated. Phosphorylation status of indicated sites was assessed by immunoblot. Immunoblots were quantified by densitometry and normalised to insulin-treated control cells. N = 2, mean ± S.E.M. Figure S2 (A and B) Total (A) and specific (B) ceramide species quantified in HeLa cells treated for 24 h with Saclac (2uM for 24 h) or vehicle control (EtOH, Control) as indicated. N = 4, mean ± S.E.M. **p< 0.01, ****p< 0.0001 vs control C) CoQ10 levels in the mitochondrial fraction obtained from HeLa cells exposed to different concentrations of Saclac or vehicle control. N = 4, mean ± S.E.M. *p<0.05 D) CoQ10 levels in the mitochondrial fraction obtained from HeLa cells exposed to different concentrations of Saclac (2 μM for 24 h) or control in presence or absence of myriocin (10 μM for 16 h). N = 4, mean ± S.E.M. *p<0.05. E-G) Mitochondrial abundance markers determined by Western Blot in HeLA cells exposed to 2 uM of Saclac for 24 h. F - G) Immunoblots were quantified by densitometry and normalised to control cells. N = 3, Mean ± S.E.M H) Percentage of non-viable HeLa cells determined by propidium iodide (PI) staining and microscopy, following a 24 h treatment with Sacl [file NIHPP2023.03.10.532020V2-supplement-1.pdf]

bioRxiv preprint doi: https://doi.org/10.1101/2023.03.10.532020; this version posted September 19, 2023. The copyright holder for this preprint (which was not certified by peer review) is the author/funder, who has granted bioRxiv a license to display the preprint in perpetuity. It is made available under aCC-BY 4.0 International license.

Supplementary figure 1

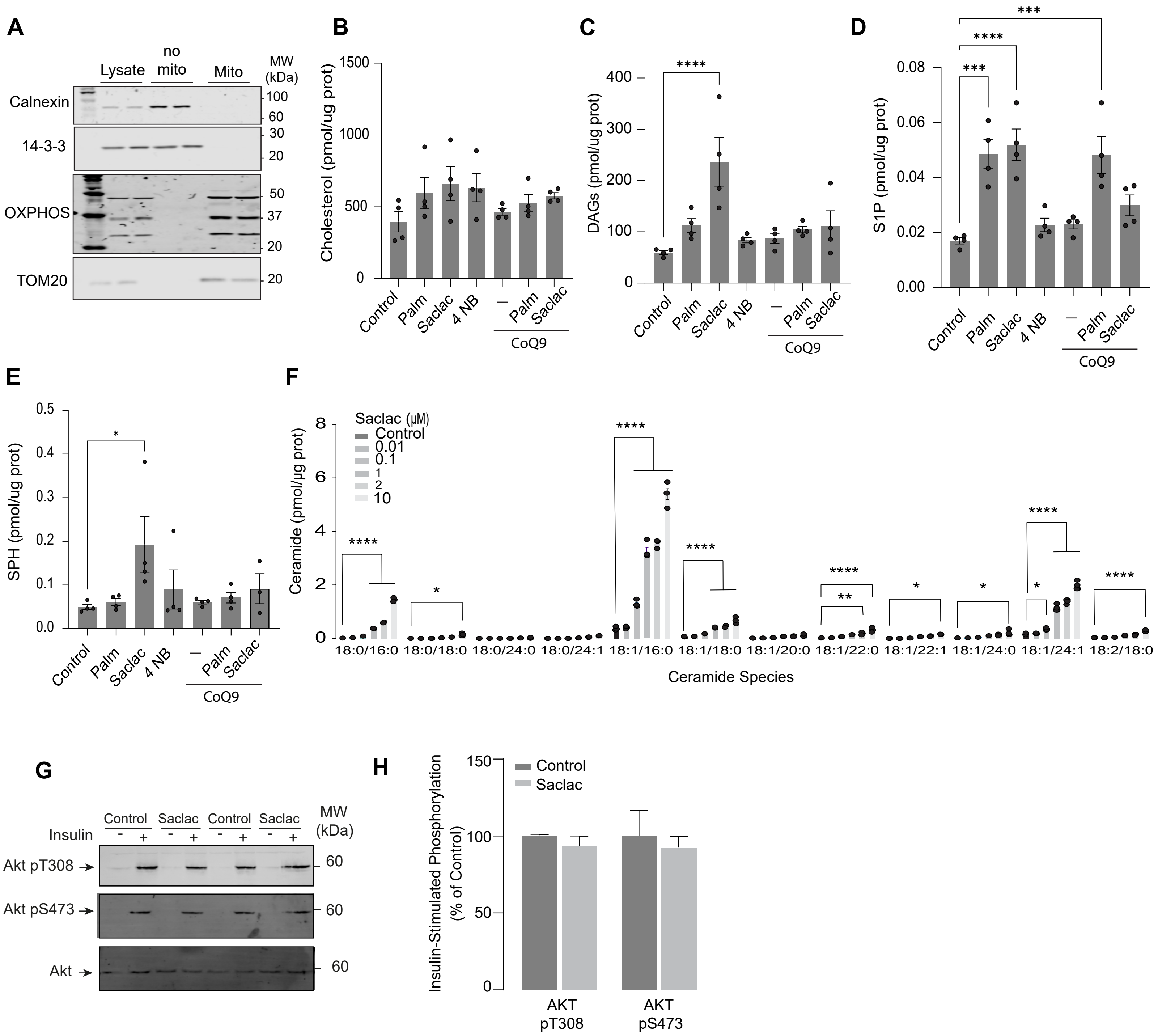

bioRxiv preprint doi: https://doi.org/10.1101/2023.03.10.532020; this version posted September 19, 2023. The copyright holder for this preprint (which was not certified by peer review) is the author/funder, who has granted bioRxiv a license to display the preprint in perpetuity. It is made available under aCC-BY 4.0 International license.

Supplementary figure 2

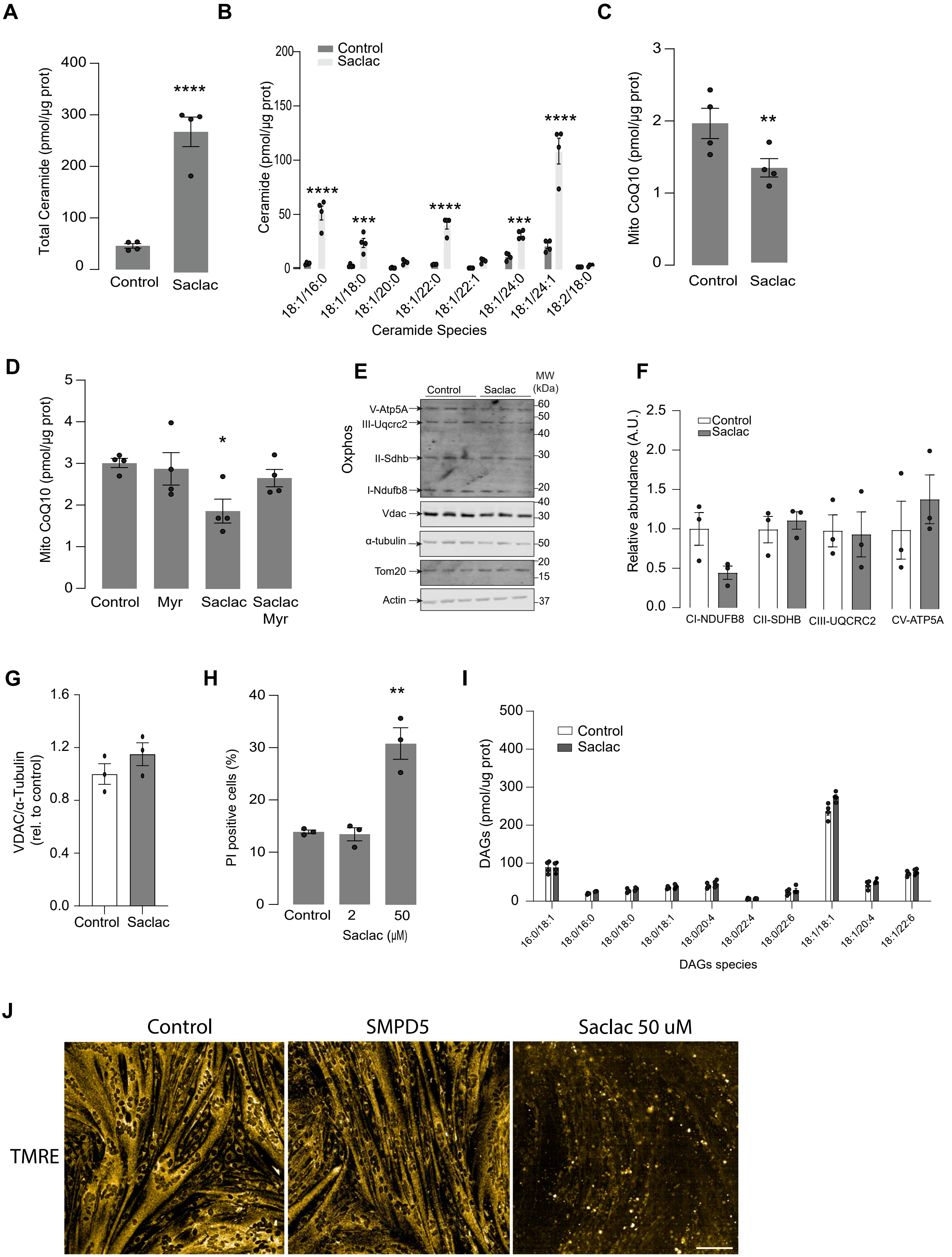

bioRxiv preprint doi: https://doi.org/10.1101/2023.03.10.532020; this version posted September 19, 2023. The copyright holder for this preprint (which was not certified by peer review) is the author/funder, who has granted bioRxiv a license to display the preprint in perpetuity. It is made available under aCC-BY 4.0 International license.

Supplementary figure 3

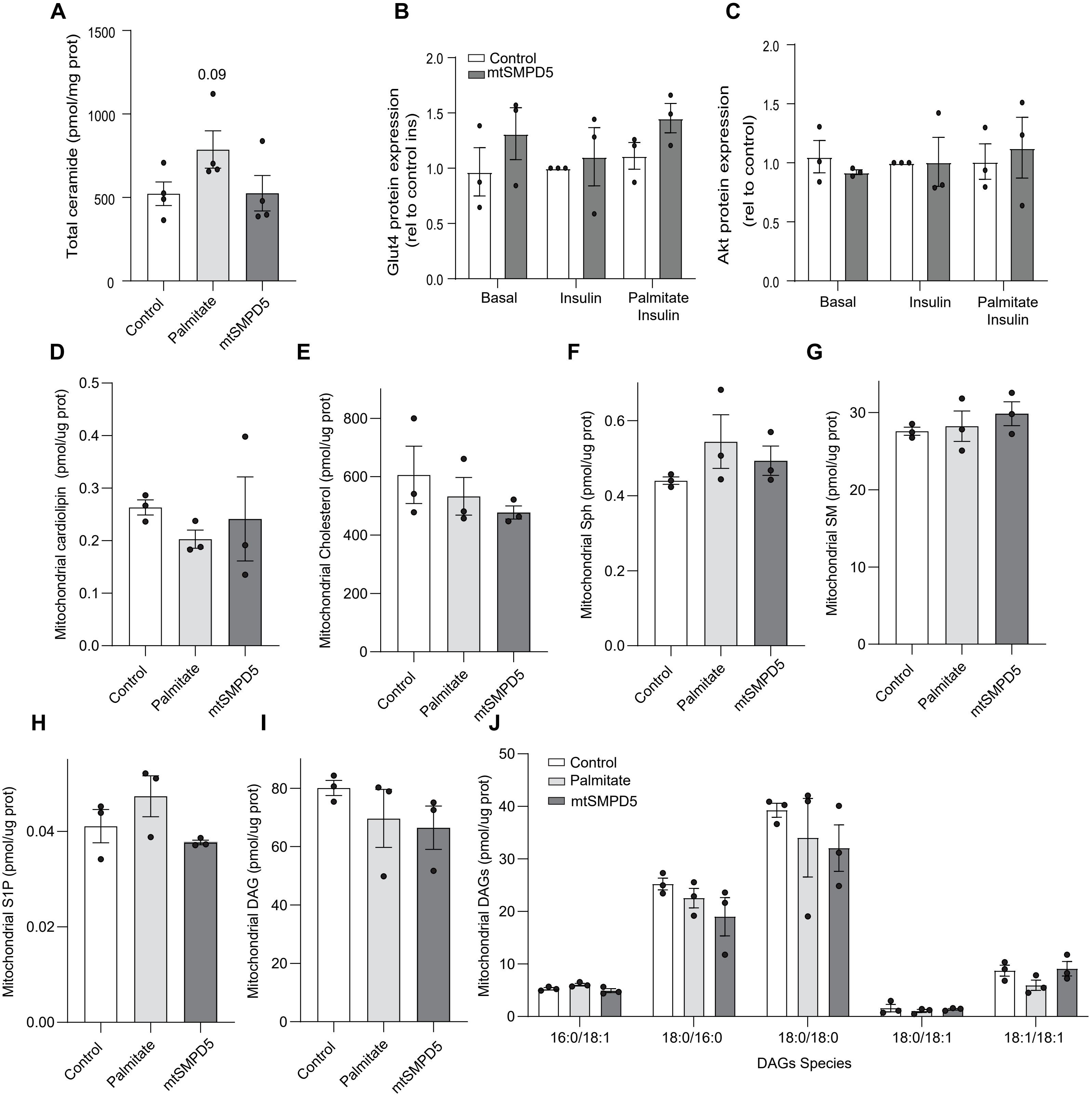

Supplementary figure 4

bioRxiv preprint doi: https://doi.org/10.1101/2023.03.10.532020; this version posted September 19, 2023. The copyright holder for this preprint (which was not certified by peer review) is the author/funder, who has granted bioRxiv a license to display the preprint in perpetuity. It is made available under aCC-BY 4.0 International license.

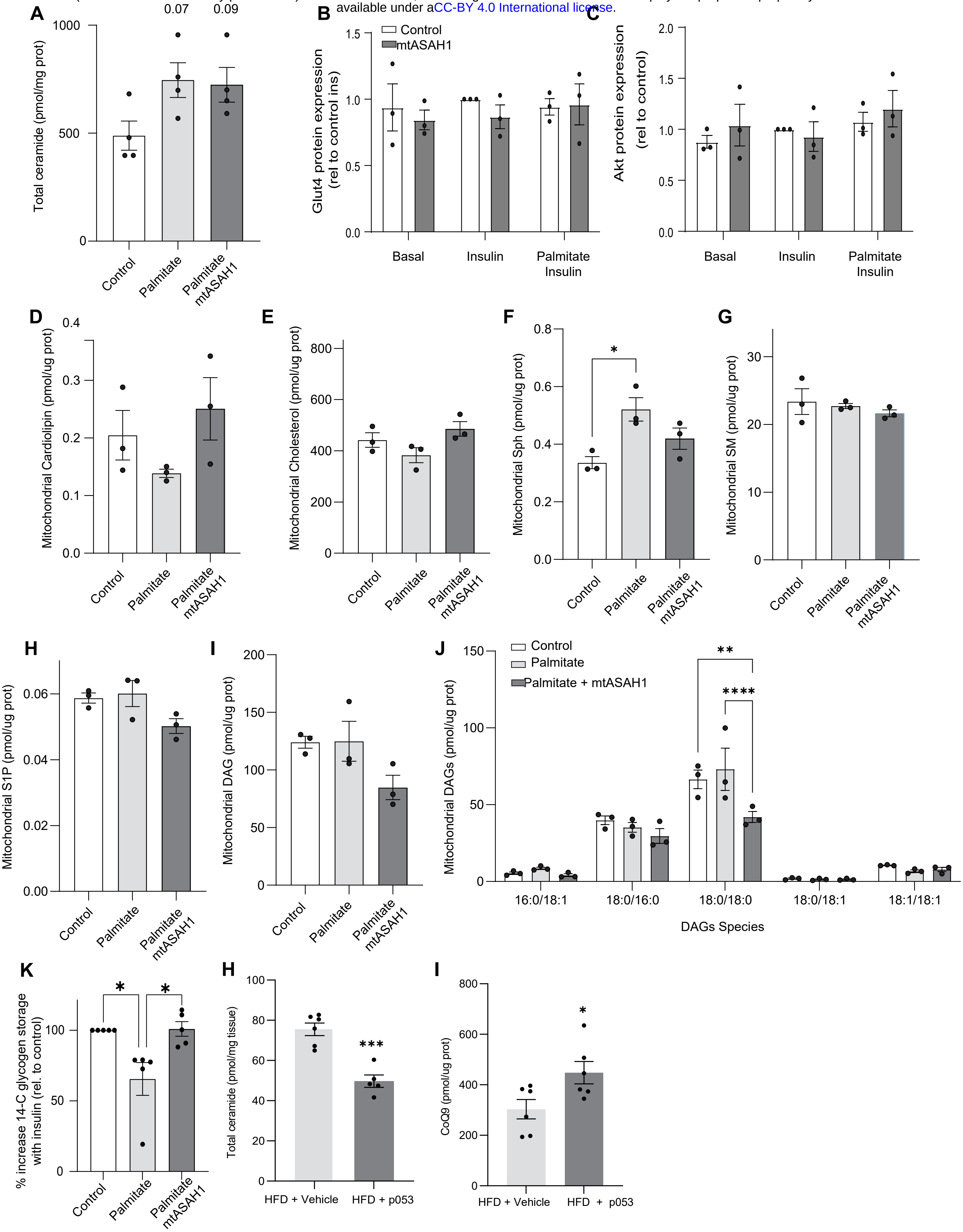

bioRxiv preprint doi: https://doi.org/10.1101/2023.03.10.532020; this version posted September 19, 2023. The copyright holder for this preprint (which was not certified by peer review) is the author/funder, who has granted bioRxiv a license to display the preprint in perpetuity. It is made available under aCC-BY 4.0 International license.

Supplementary figure 5

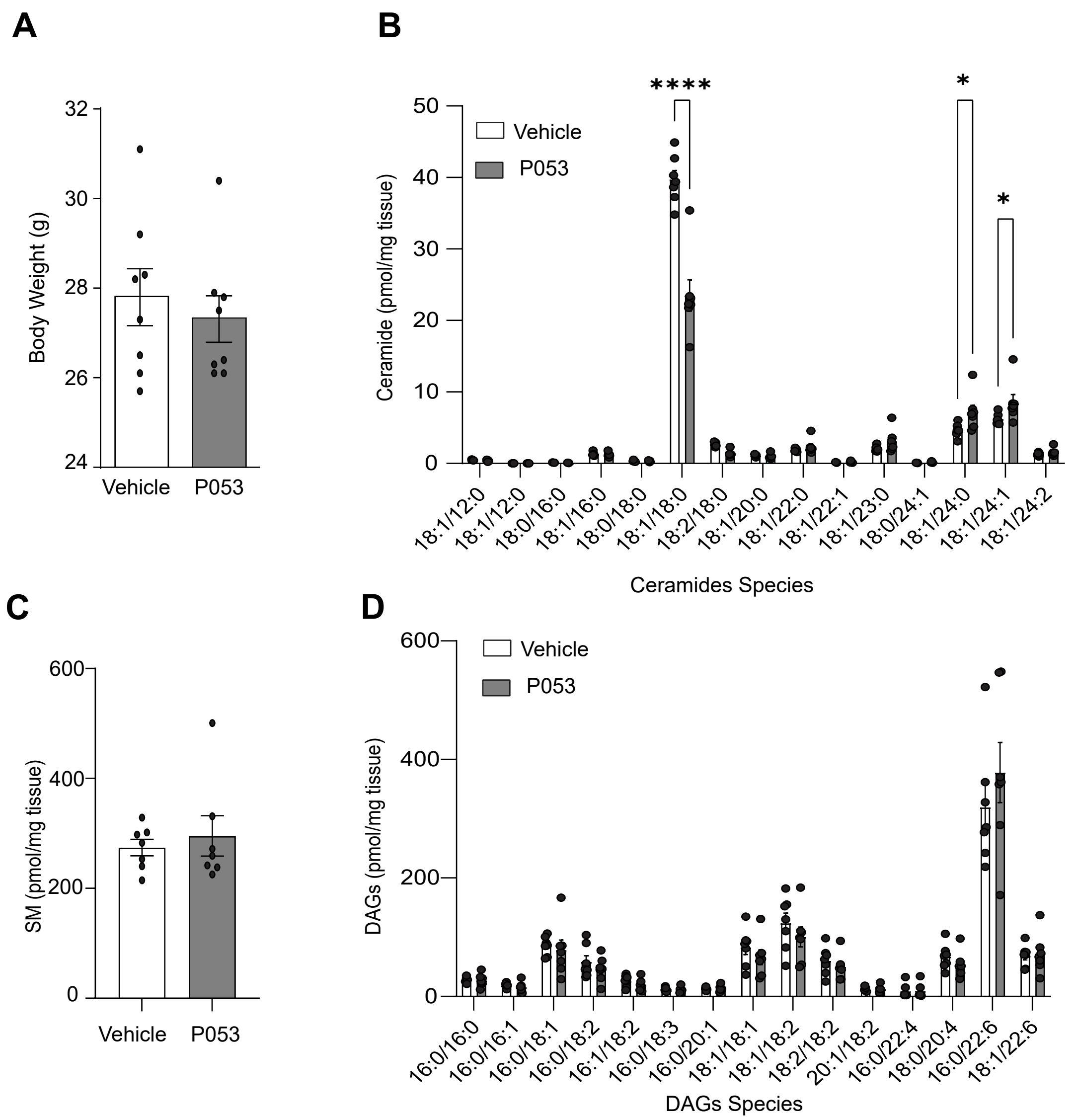

bioRxiv preprint doi: https://doi.org/10.1101/2023.03.10.532020; this version posted September 19, 2023. The copyright holder for this preprint (which was not certified by peer review) is the author/funder, who has granted bioRxiv a license to display the preprint in perpetuity. It is made available under aCC-BY 4.0 International license.

Supplementary figure 6

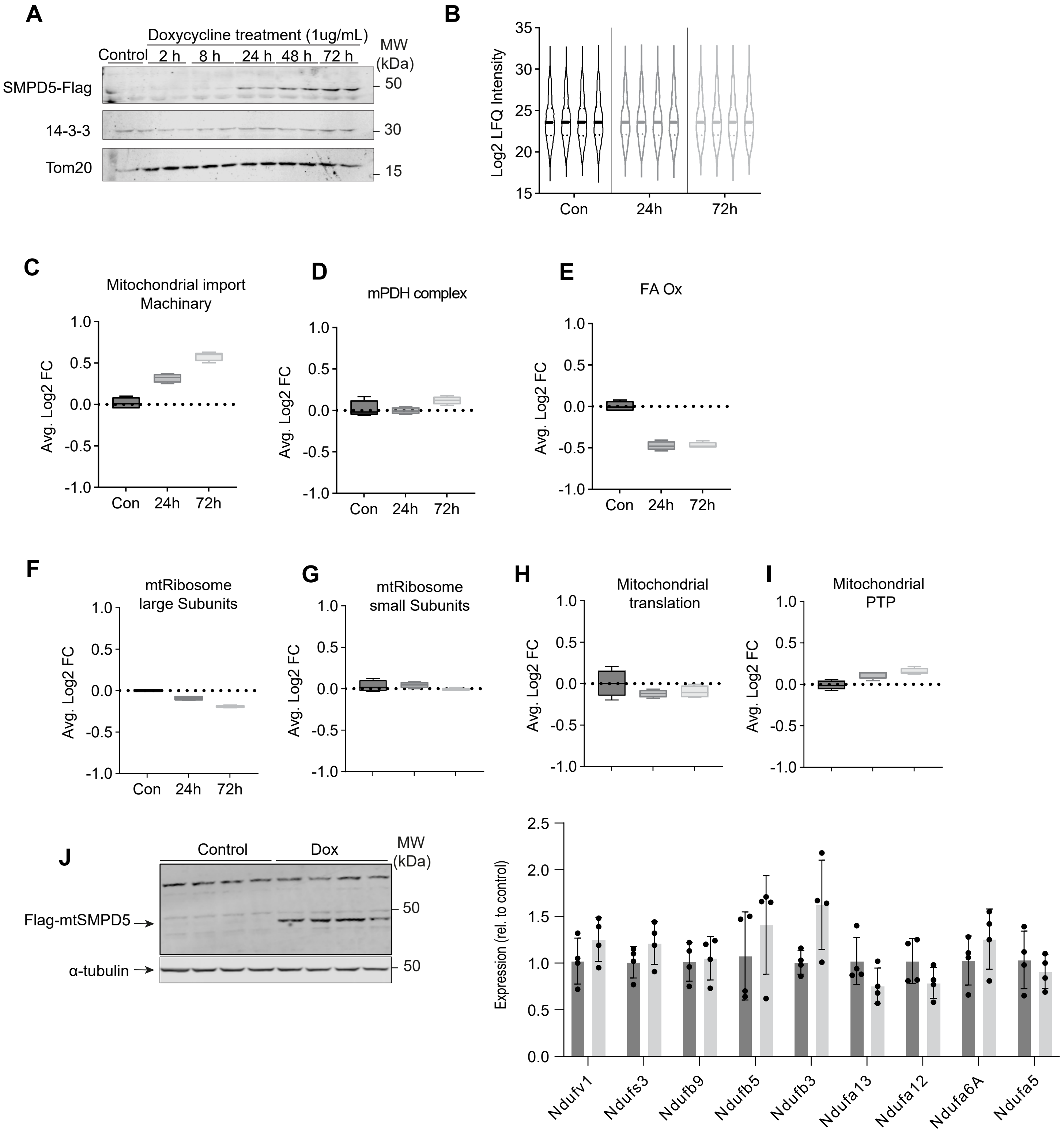

bioRxiv preprint doi: https://doi.org/10.1101/2023.03.10.532020; this version posted September 19, 2023. The copyright holder for this preprint (which was not certified by peer review) is the author/funder, who has granted bioRxiv a license to display the preprint in perpetuity. It is made available under aCC-BY 4.0 International license.

Supplementary figure 7

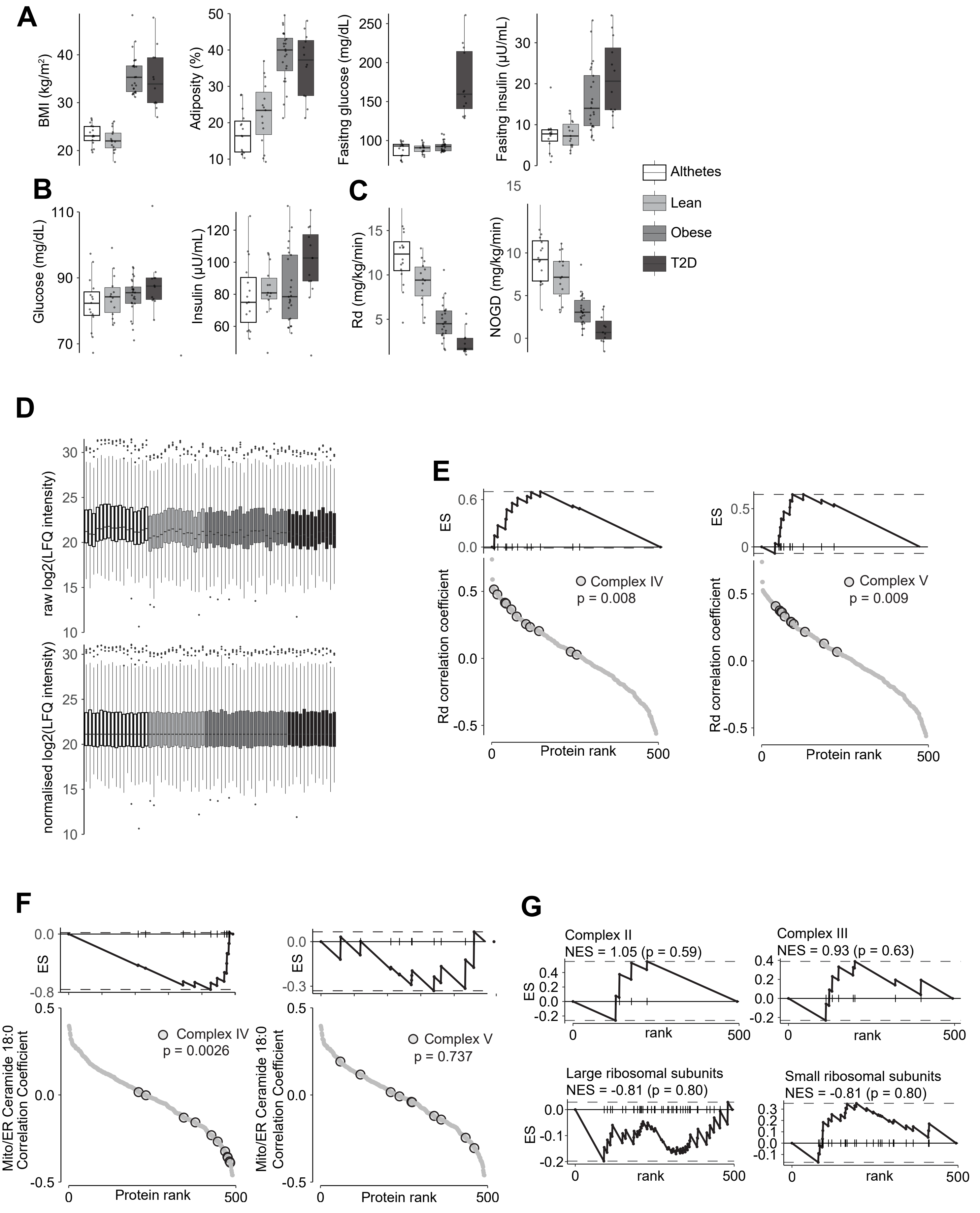

bioRxiv preprint doi: https://doi.org/10.1101/2023.03.10.532020; this version posted September 19, 2023. The copyright holder for this preprint (which was not certified by peer review) is the author/funder, who has granted bioRxiv a license to display the preprint in perpetuity. It is made available under aCC-BY 4.0 International license.

Supplementary figure 8

Oligo Name	Sequence
rNdufa10_R	AAAGCCCGTACCTCAGCTTG
rNdufa12_F	CTGGGATGTGGATGGAAGCA
rNdufa12_R	TGTTGTTGGAGGGTCGTCAG
rNdufa13_F	ATGGGTTACGCACCAAGGAG
rNdufa13_R	ATTATGCACGTCGGCTCAGG
rNdufa5_F	TCTACGTTCGATTGAGCGGG
rNdufa5_R	CAATCCCACCAGGCCAGTAG
rNdufa6_F	CGGGGCTTCCTTAGCAAGAT
rNdufa6_R	GTCCCGACTGAAAATGGGCT
rNdufb3_F	GTGTCTCCTACCGCAGTCAA
rNdufb3_R	GCAAGGCTCCCAGACAAGAC
rNdufb5_F	TGTGGTTCAGGTGATGCGTT
rNdufb5_F	AGTTCCCAGGCCTCTAGCTT
rNdufv1_F	ACCTCATTTGGCTCGCTGAA
rNdufv1_R	CCTTCAGCCTCCAGTCATGG
rNdufs3_F	ATTTCCACTTCCGGTCCGTG
rNdufs3_R	CATGTTCCTTAGGGTGCCGA
rNdufb9_F	GCTTGATGAGAGCCCGGTTT
rNdufb9_F	TGAGGATGCTGGTTTTGCCA
