# Supplementary material for: Mitochondrial electron transport chain, ceramide and Coenzyme Q are linked in a pathway that drives insulin resistance in skeletal muscle
Source: bioRxiv. 2023 Mar 11:2023.03.10.532020. Preprint. [Version 1] doi: 10.1101/2023.03.10.532020 (PMC10028964; doi:10.1101/2023.03.10.532020)
Supplement: Supplementary file 1 [file NIHPP2023.03.10.532020v1-supplement-1.pdf]

bioRxiv preprint doi: https://doi.org/10.1101/2023.03.10.532020; this version posted March 12, 2023. The copyright holder for this preprint (which was not certified by peer review) is the author/funder, who has granted bioRxiv a license to display the preprint in perpetuity. It is made available under aCC-BY 4.0 International license.

Supplementary figure 1

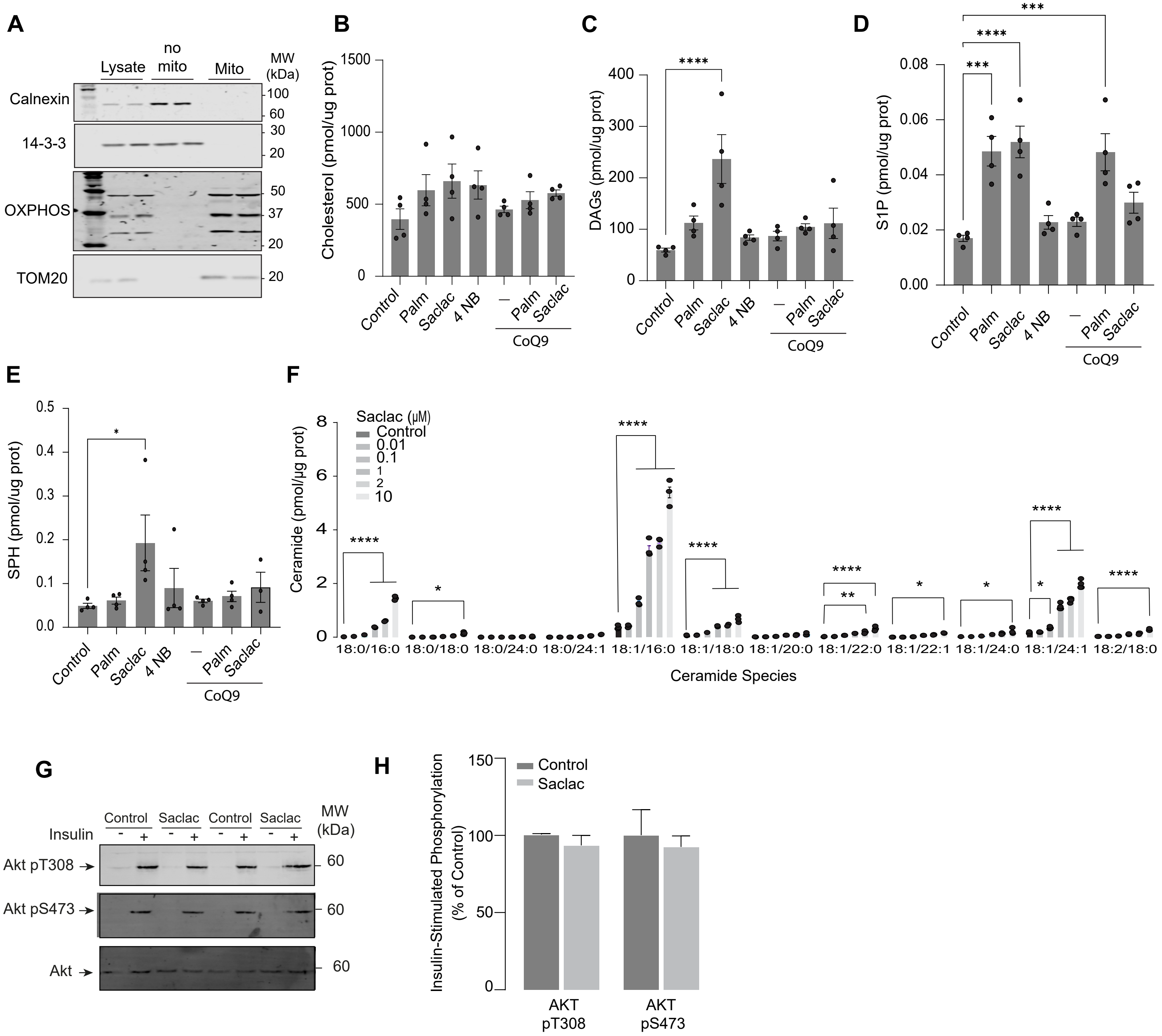

bioRxiv preprint doi: https://doi.org/10.1101/2023.03.10.532020; this version posted March 12, 2023. The copyright holder for this preprint (which was not certified by peer review) is the author/funder, who has granted bioRxiv a license to display the preprint in perpetuity. It is made available under aCC-BY 4.0 International license.

Supplementary figure 2

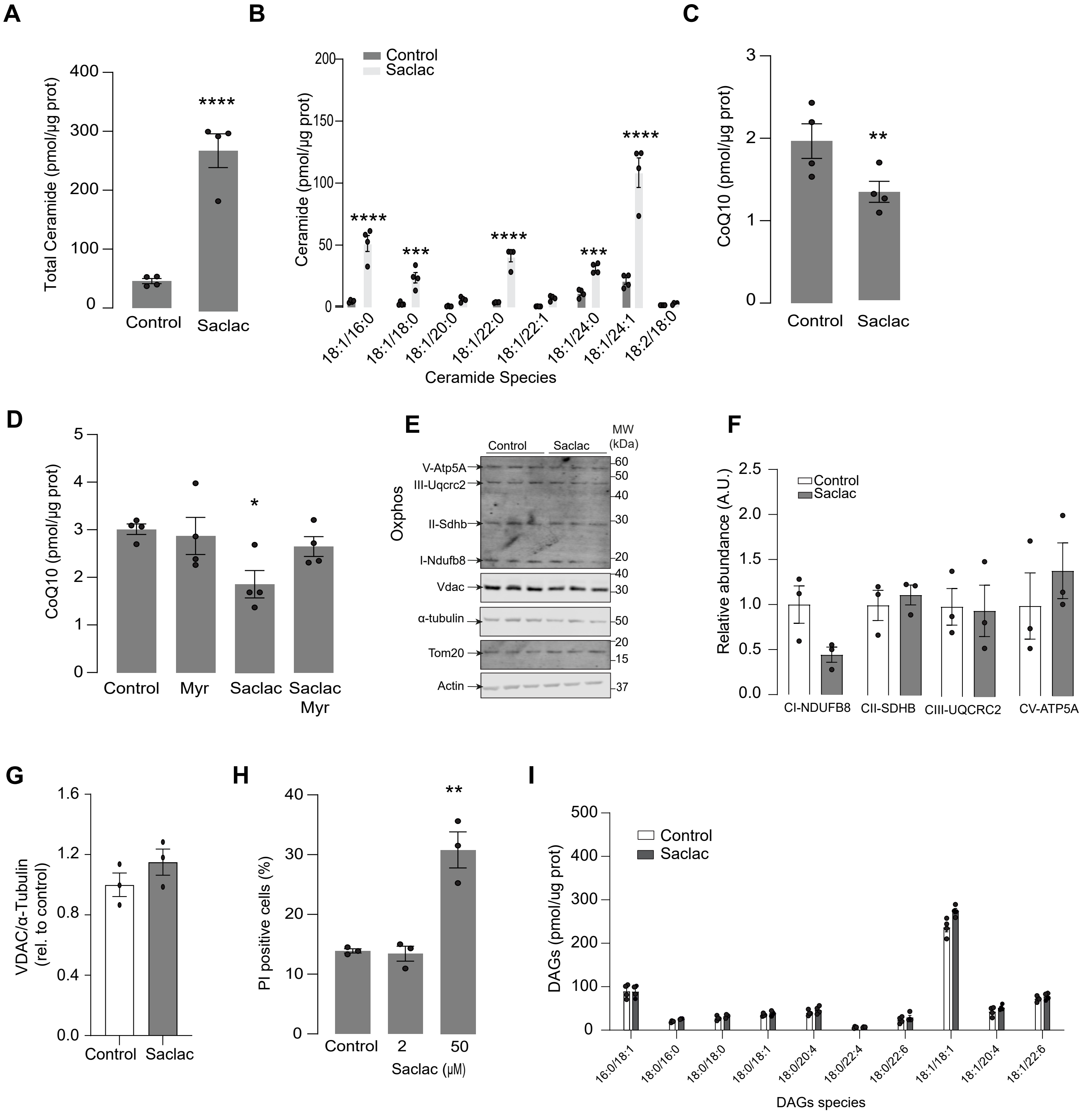

bioRxiv preprint doi: https://doi.org/10.1101/2023.03.10.532020; this version posted March 12, 2023. The copyright holder for this preprint (which was not certified by peer review) is the author/funder, who has granted bioRxiv a license to display the preprint in perpetuity. It is made available under aCC-BY 4.0 International license.

Supplementary figure 3

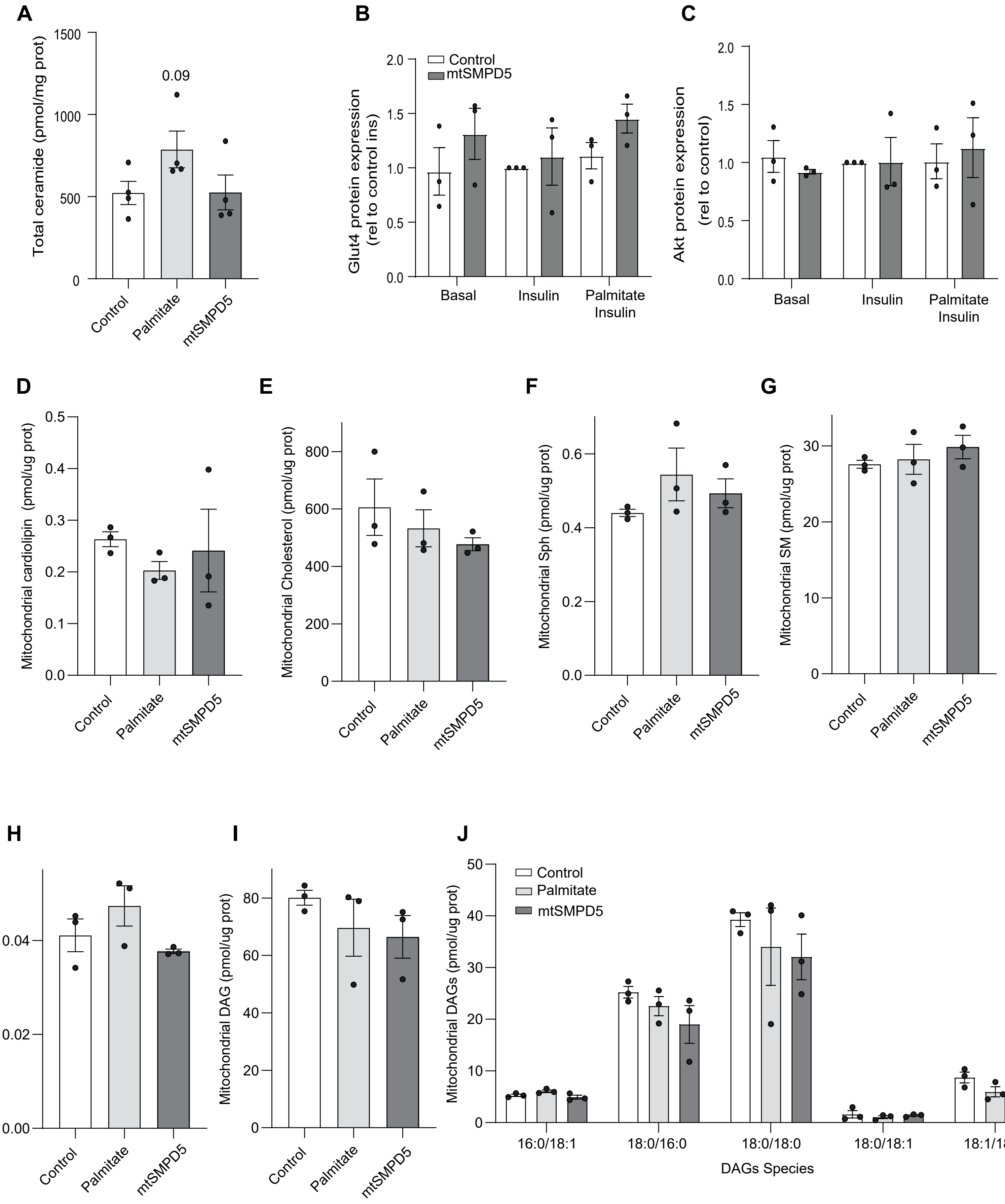

bioRxiv preprint doi: https://doi.org/10.1101/2023.03.10.532020; this version posted March 12, 2023. The copyright holder for this preprint (which was not certified by peer review) is the author/funder, who has granted bioRxiv a license to display the preprint in perpetuity. It is made available under aCC-BY 4.0 International license.

Supplementary figure 4

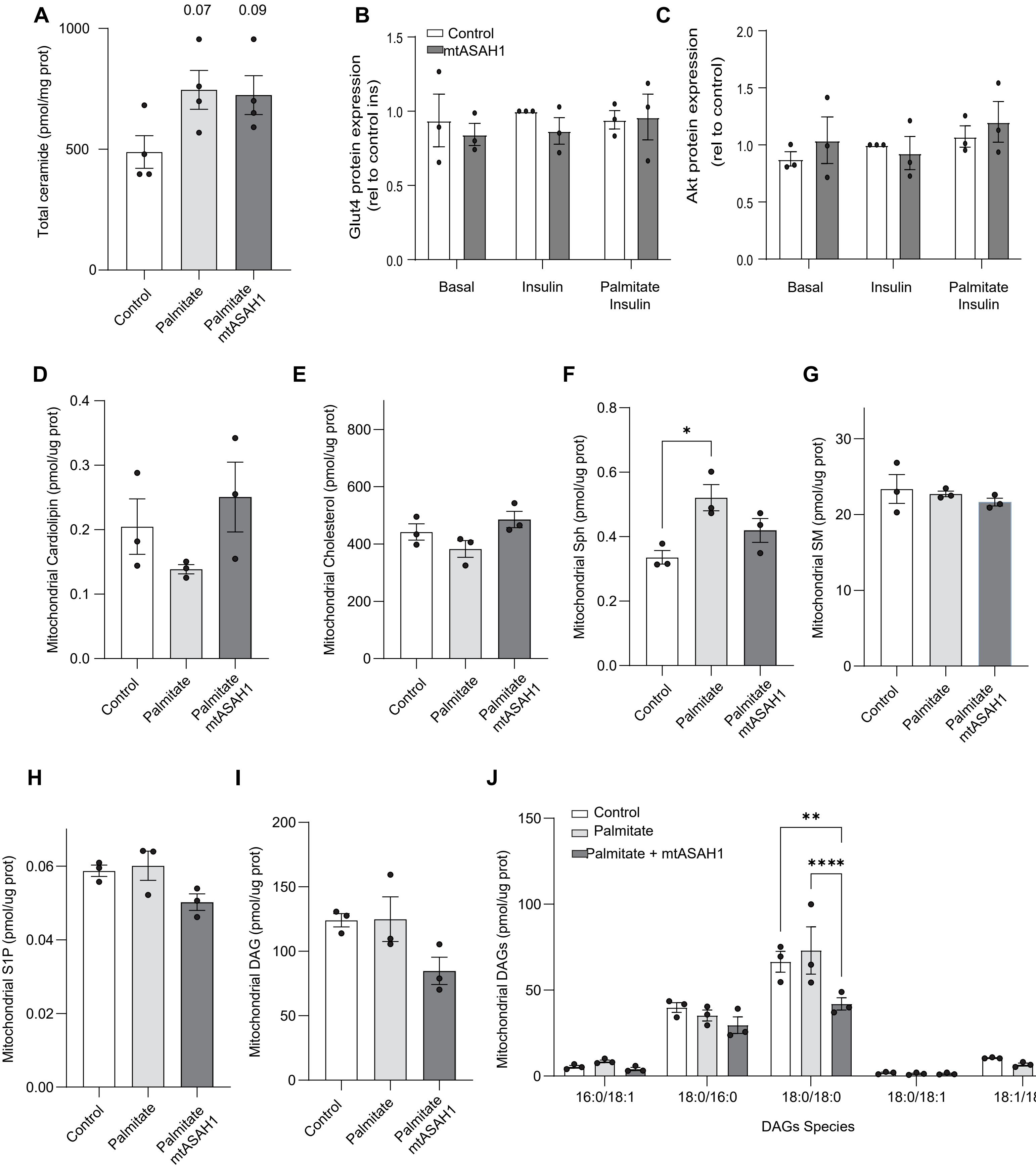

bioRxiv preprint doi: https://doi.org/10.1101/2023.03.10.532020; this version posted March 12, 2023. The copyright holder for this preprint (which was not certified by peer review) is the author/funder, who has granted bioRxiv a license to display the preprint in perpetuity. It is made available under aCC-BY 4.0 International license.

Supplementary figure 5

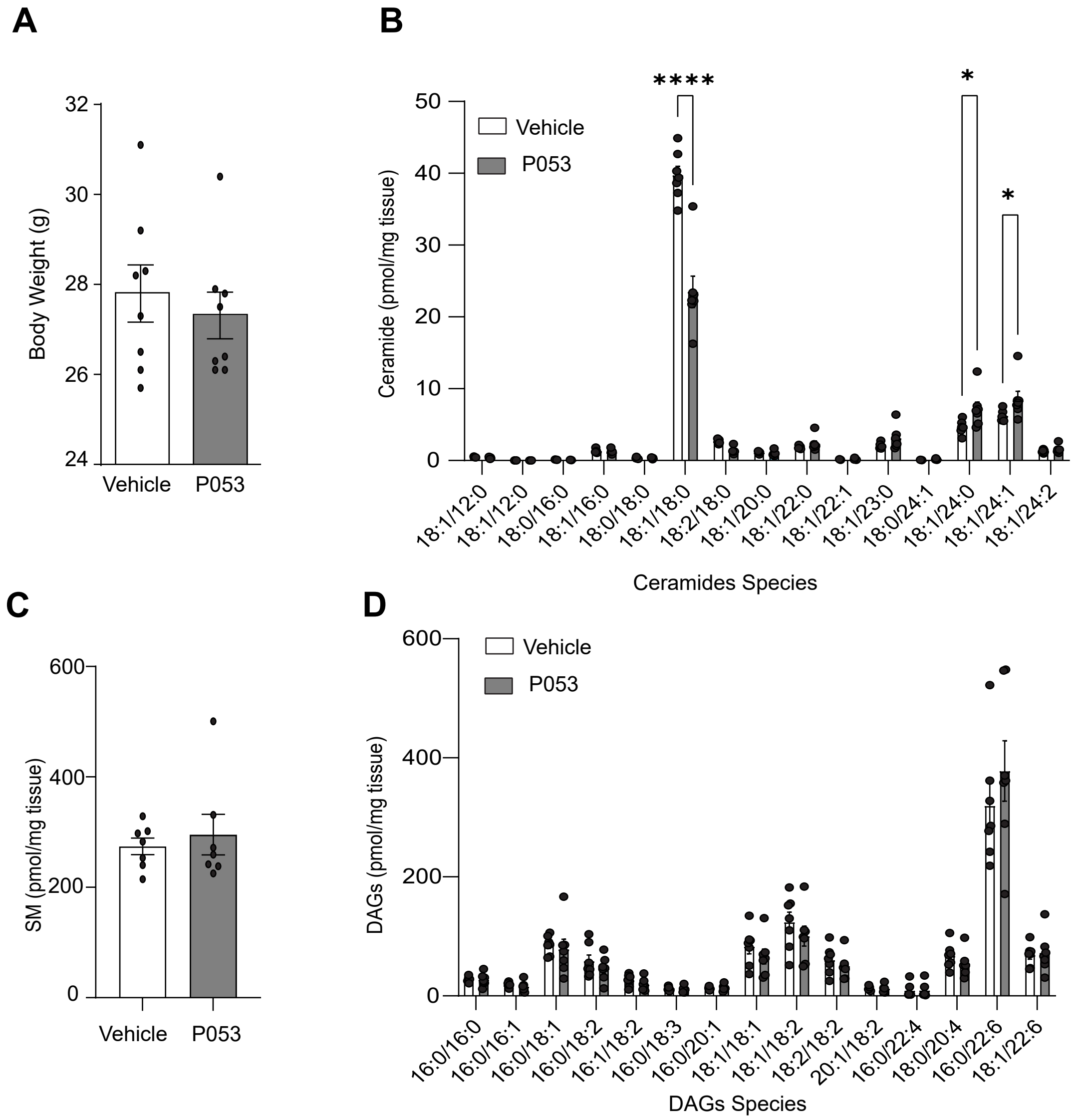

bioRxiv preprint doi: https://doi.org/10.1101/2023.03.10.532020; this version posted March 12, 2023. The copyright holder for this preprint (which was not certified by peer review) is the author/funder, who has granted bioRxiv a license to display the preprint in perpetuity. It is made available under aCC-BY 4.0 International license.

Supplementary figure 6

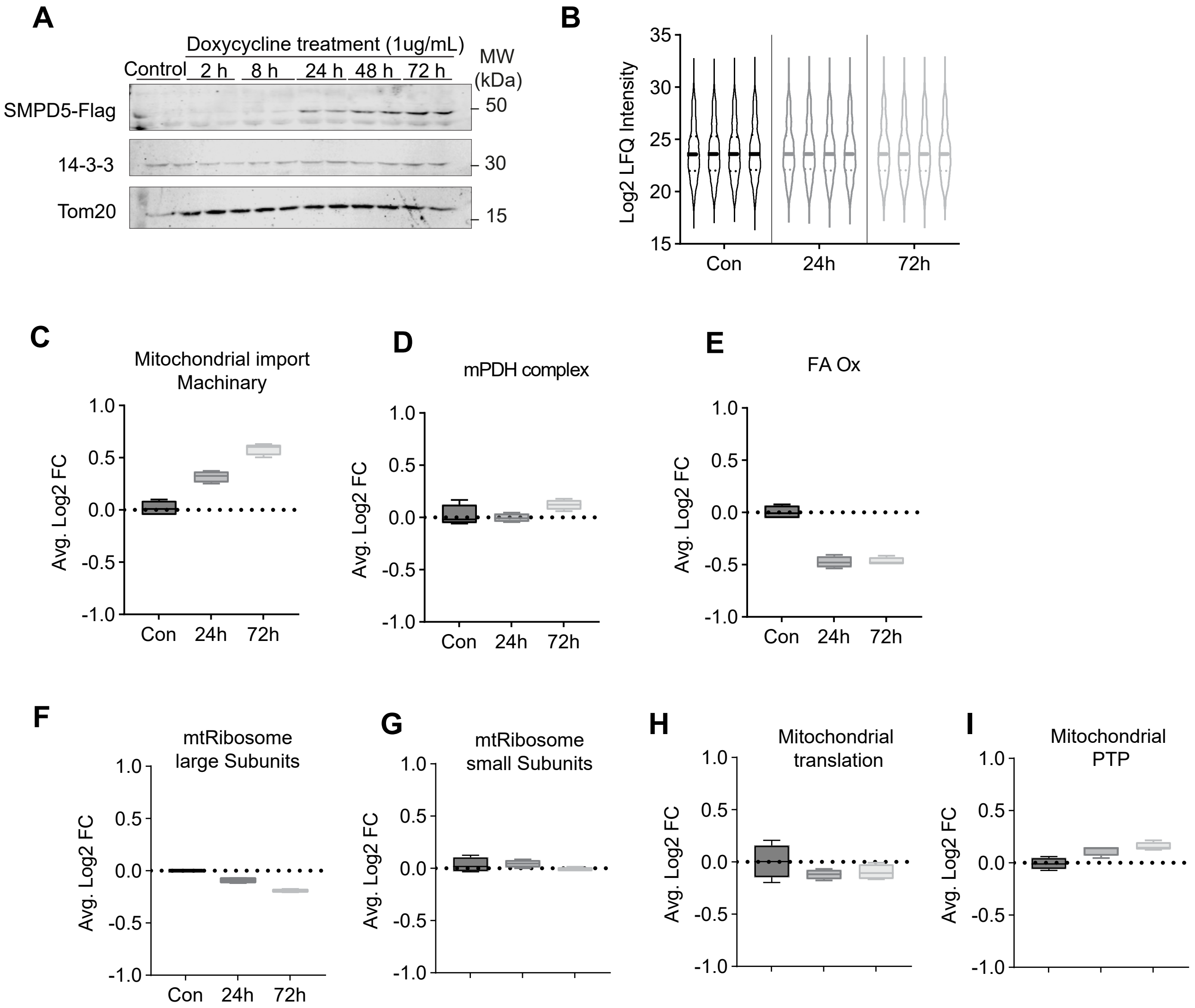

bioRxiv preprint doi: https://doi.org/10.1101/2023.03.10.532020; this version posted March 12, 2023. The copyright holder for this preprint (which was not certified by peer review) is the author/funder, who has granted bioRxiv a license to display the preprint in perpetuity. It is made available under aCC-BY 4.0 International license.

Supplementary figure 7

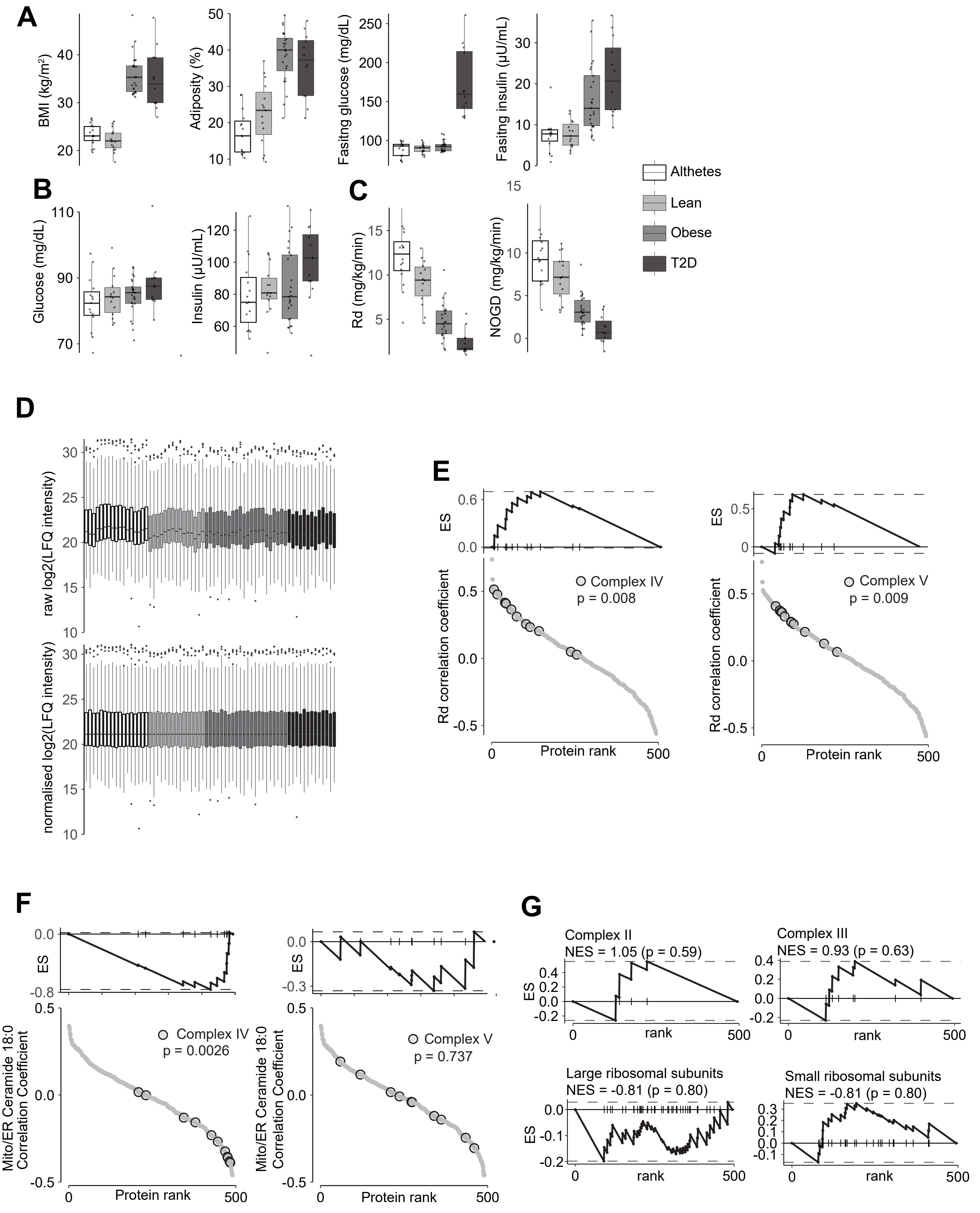

bioRxiv preprint doi: https://doi.org/10.1101/2023.03.10.532020; this version posted March 12, 2023. The copyright holder for this preprint (which was not certified by peer review) is the author/funder, who has granted bioRxiv a license to display the preprint in perpetuity. It is made available under aCC-BY 4.0 International license.
